# The effect of curcumin on anthropometric indices, blood pressure, lipid profiles, fasting blood glucose, liver enzymes, fibrosis, and steatosis in non-alcoholic fatty livers

**DOI:** 10.3389/fnut.2023.1163950

**Published:** 2023-05-18

**Authors:** Zahra Safari, Mohammad Bagherniya, Ziba Khoram, Amrollah Ebrahimi Varzaneh, Zahra Heidari, Amirhossein Sahebkar, Gholamreza Askari

**Affiliations:** ^1^Nutrition and Food Security Research Center and Department of Community Nutrition, School of Nutrition and Food Science, Isfahan University of Medical Sciences, Isfahan, Iran; ^2^Anesthesia and Critical Care Research Center, Isfahan University of Medical Sciences, Isfahan, Iran; ^3^Gastroenterology and Hepatology Research Center, Isfahan University of Medical Sciences, Isfahan, Iran; ^4^Department of Biostatistics and Epidemiology, School of Health, Isfahan University of Medical Sciences, Isfahan, Iran; ^5^Isfahan Cardiac Rehabilitation Research Center, Cardiovascular Research Institute, Isfahan University of Medical Sciences, Isfahan, Iran; ^6^Biotechnology Research Center, Pharmaceutical Technology Institute, Mashhad University of Medical Sciences, Mashhad, Iran; ^7^Applied Biomedical Research Center, Mashhad University of Medical Sciences, Mashhad, Iran; ^8^School of Medicine, The University of Western Australia, Perth, WA, Australia

**Keywords:** non-alcoholic fatty liver, curcumin, fibrosis, steatosis, FibroScan

## Abstract

**Background:**

Non-alcoholic fatty liver disease (NAFLD) is the most common form of liver disease. Curcumin is a natural polyphenol that may be effective against liver steatosis and steatohepatitis. The present study aimed to evaluate the effects of phytosomal curcumin on lipid profile, fasting blood sugar, anthropometric indices, liver enzymes, fibrosis, and steatosis in non-alcoholic fatty liver patients.

**Methods:**

The participants were randomized to the curcumin–phosphatidylserine phytosomal receiving group and the placebo receiving group and were followed up for 12 weeks. Data on anthropometric indices, lipid profile, blood glucose, blood pressure, liver enzymes, hepatic steatosis, and fibrosis were collected at the beginning and the end of the clinical trial.

**Results:**

Supplementation for 12 weeks with phytosomal curcumin significantly reduced fibrosis and steatosis in the phytosomal curcumin receiving group compared with the placebo group (*p* < 0.05). Phytosomal curcumin also significantly reduced waist circumference and blood pressure compared with the placebo group (*p* < 0.05). There was no significant difference between the phytosomal curcumin and the placebo groups regarding changes in weight, body mass index, fasting blood glucose, liver enzymes, and lipid profile.

**Conclusion:**

Curcumin, at a dose of 250 mg per day, might be effective in treating patients with NAFLD. Further studies are necessary to confirm these findings and to discover the underlying mechanisms.

**Clinical trial registration:**

https://www.irct.ir/trial/43730, identifier: IRCT20121216011763N39.

## Introduction

Non-alcoholic fatty liver disease (NAFLD) is one of the most common chronic liver disorders with a prevalence of over 20% globally, which is mostly due to the lifestyle changes such as increased inactivity and switching to high-fat and high-calorie diets (1). NAFLD is significantly associated with cardiometabolic risk factors such as obesity, hypertension, insulin resistance, type 2 diabetes, and dyslipidemia, which are components of metabolic syndrome (2).

Insulin resistance is a major pathophysiological factor for metabolic syndrome, independently associated with hepatic steatosis (3). Oxidative stress, on the one hand, is defined as an imbalance between prooxidant species and antioxidants. On the other hand, it is an important factor in the progression of fatty liver, which is associated with inflammation and lipotoxicity (4).

The diagnosis of NAFLD is confirmed when simple steatosis develops without other causes of liver disease such as viral hepatitis, autoimmune diseases, alcohol or drug use, and alpha-1 antitrypsin deficiency (5). The use of non-invasive methods such as ultrasound and FibroScan to confirm steatosis of the liver has been approved (6). FibroScan has an 88% sensitivity for diagnosing advanced fibrosis (7), and its main advantage is the possibility of measuring steatosis and fibrosis (8).

To date, no exclusive treatment for NAFLD is known (9, 10). Previous studies have shown that diets rich in antioxidants and anti-inflammatory agents, such as active ingredients in some herbs, can be effective in treating NAFLD (11). One of these substances is curcumin, which is the active ingredient of turmeric with numerous identified salutary benefits including antioxidant (12), antimicrobial (13), antifungal (14), antiviral (15), anti-inflammatory (16), and antitumor properties (17–21) and can play a vital role in preventing liver fibrosis (22). The antioxidant and anti-inflammatory properties of curcumin are responsible for neutralizing free radicals and inhibiting the production of prostaglandins (23) as well as proinflammatory cytokines (24, 25). Conversely, curcumin has antidiabetic properties and affects fatty liver disease by reducing insulin resistance (26).

Despite favorable effects in preclinical models, the clinical use of curcumin might be hampered by its low oral bioavailability. Therefore, several solutions have been put forward to enhance the bioavailability of this phytochemical by harnessing drug delivery systems. Phytosomes are liposome-like vesicles that have been suggested as the carrier systems for plant bioactive compounds (27). The phospholipids used in the production of phytosomes not only serve as a carrier but also play a protective role for the liver (28, 29). Moreover, phosphatidylserine in phytosomal curcumin enhances the anti-inflammatory properties of curcumin (30). Phytosomes not only protect plant bioactive compounds from the effects of gastrointestinal degrading factors but also have a controllable release profile. Furthermore, few studies have been performed to investigate the phytosomal curcumin effect on fatty liver. Therefore, further studies are necessary to draw conclusions based on the positive or non-positive effect of the phytosomal curcumin on the treatment of non-alcoholic fatty liver by the non-invasive FibroScan method.

## Materials and methods

### Study design and participants

The current study was a double-blind, parallel, placebo-controlled, randomized clinical trial to assess the effect of phytosomal curcumin on NAFLD. The study protocol was approved by the ethics committee of the Isfahan University of Medical Sciences, Isfahan (No. IR.MUI.RESEARCH.REC.1398.461). All participants provided written informed consent. The trial was registered at the Iranian Registry of Clinical Trial (No. IRCT20121216011763N39).

Participants were selected from the outpatients who were referred to the clinics affiliated with Isfahan University of Medical Sciences from November 2020 to April 2021. The inclusion criteria were the willingness to participate in the study; age between 18 and 65 years; and non-alcoholic fatty liver disease (grades 1–3) diagnosed by previous ultrasound. The exclusion criteria include pregnancy and lactation, alcoholic fatty liver disease, smoking, heart, lung, kidney, hepatitis, cirrhosis, biliary and immune system disorders, diabetes, Cushing's syndrome, the use of lipid and blood sugar lowering drugs, supplementation with vitamin E and other antioxidants, ursodeoxycholic acid, phenytoin, tamoxifen, lithium, corticosteroids and methotrexate, weight loss, and bariatric surgery in the last year. Also, participants who were unwilling to continue treatment or had poor adaptation to trial therapy (80%) were excluded from the trial during the follow-up period.

The randomization method was performed using a computer randomization sequence in permuted blocks of four, in which patients were classified based on gender and steatosis status. They were assigned to receive either phytosomal curcumin or placebo capsules for 12 weeks. Researchers, patients, and statistical consultants were all blinded. The placebo capsule was identical to the curcumin capsule in shape, color, size, odor, and weight. All capsules were distributed in similar containers.

### Intervention

Patients in the curcumin group took one capsule containing phytosomal curcumin (250 mg containing 20% curcuminoids and 20% phosphatidylserine; Indena S.p.A, Milan, Italy) daily (curcuminoids are the natural polyphenols present in Curcuma longa rhizome; in particular, Meriserin^®^ contains all three most potent turmeric curcuminoids, i.e., curcumin, demethoxycurcumin, and bisdemethoxycurcumin) and patients in the placebo group took one placebo capsule (which contains 250 mg of maltodextrin) daily after breakfast for 12 weeks. All patients received the same dietary recommendations for fatty liver and physical activity. Patients' compliance was assessed by counting the returned capsules.

### Outcome assessment

The primary outcome of this study was a reduction in steatosis and hepatic fibrosis. The secondary outcomes were changes in fasting blood sugar, lipid profile, liver enzymes, hypertension, and anthropometric indices.

At baseline, demographic characteristics, including age, height, weight, marital status, blood glucose, and lipid-lowering drugs such as statins and metformin, were recorded using a general questionnaire.

Blood samples were taken from the cubital vein after one night of fasting. Blood serum was collected and analyzed immediately. Fasting blood glucose (FBG), alanine aminotransferase (ALT), aspartate aminotransferase (AST), total cholesterol (TC), and triglyceride (TG) were assessed by enzymatic methods using Pars Azmun Kits (Pars Azmun, Tehran, Iran). The serum concentration of low-density lipoprotein cholesterol (LDL) was calculated using the Friedwald formula: LDL – C = TC – HDL – 0.16 [TG] (31). In addition, the concentration of high-density lipoprotein (HDL) was assessed by enzymatic methods using the Biorex Fars kit (Biorex Fars, Shiraz, Iran).

Anthropometric measurements were attained with standardized equipment and procedures. Weight was measured using a digital balance (seca 831, Hamburg, Germany) to the nearest 0.1 kg with an item of light clothing. The height of the participants was measured without shoes to the nearest 0.1 cm by a wall-mounted stadiometer (seca 206, Hamburg, Germany); the body mass index (BMI) was calculated as the weight-to-height squared ratio; and the waist circumference (WC) was measured by an inelastic meter.

The patient's blood pressure was taken after the patient remained sitting for at least 5 min. The patient's blood pressure was measured twice and finally averaged at each visit.

To evaluate the nutritional status of patients, 3 days of food records (2 weekdays and 1 holiday) were obtained at the start of the study, at the sixth week, and at the end of the study; the average of 3 days was calculated and reported. Using the information obtained from these records, the amount of food intake and daily energy and calories received from food according to the Nutritionist IV software version 7.0 (N-squared Computing, Salem, OR, USA) as well as the relevant data were entered in the SPSS software version 22 (SPSS Inc., Chicago, IL, USA).

We obtained data on participants' physical activity in terms of metabolic equivalent (MET) per hour per day (MET/h/day) by requiring them to complete a 3-day physical activity registration letter. People were asked to report on their activities from walking, exercising, sleeping, watching TV, doing household work, studying, bathing, etc. The total metabolic equivalent was calculated by multiplying the frequency, duration, and intensity of each physical activity in 24 h. Then, from the recording average, the final number was reported as the MET/h/day.

#### FibroScan

Transient elastography (TE) or FibroScan is often used for liver stiffness measurement (LSM) in patients suffering from various chronic liver diseases (32). The FibroScan device (Echosens) operates by measuring the shear wave velocity. In this method, a 50-MHz wave passes through a small transducer at the end of an ultrasound probe. The probe also has a converter that can measure shear wave velocities (in m/kg). This technology was used to measure the speed of the wave that passes through the liver. The harder the texture, the faster the shear wave propagation (33). The benefits of a FibroScan are painless, fast (usually < 5 min), and carried out on the patient in bed without the need for hospitalization (34).

The procedure was performed in such a way that the patient laid down, and the right arm of the patient was placed behind the head to facilitate access to the upper quadrant of the abdomen (35). A painless mechanical shock was transmitted to the intercostal space above the liver, creating a wave of mechanical deformation (36, 37). The low-frequency ultrasound transducer controls the propagation of the wave through the limb and produces the propagation velocity that is related to the mechanical properties of the environment (38). The harder the texture, the faster the shear wave propagation. This method made it possible to estimate liver stiffness in kilopascals (kPa), which was associated with liver fibrosis. The FibroScan probe was placed perpendicular to the skin. A visual indicator standardized the pressure applied by the operator. Ten repeated measurements were performed. The final result was the middle of all valid measurements noted. The scores of FibroScan were as follows (39): 4/7 – 5 showed minimal fibrosis; 5 – 9/4 suggested moderate fibrosis; and 9/5 or higher indicated severe fibrosis or cirrhosis.

The FibroScan device used in this trial was the Touch Echosens 502 of France model with serial numbers in F 60759 and Ref 1907-100-000.

### Statistical analysis

All statistical analyses were performed using SPSS version 22 (SPSS Inc., Chicago, IL, USA). The Kolmogorov–Smirnov test evaluated the normality quantitative data. Data were presented as mean ± standard deviation (SD) for quantitative variables and as frequency (percentage) for categorical data. Independent sample *t*-tests or chi-squared tests were used to explore the bivariate differences at baseline and the end of the intervention. The changes in quantitative data were compared before and after the intervention using a paired sample *t*-test. The baseline characteristics of participants were compared between the groups by independent samples *t*-test or chi-squared test. One-way ANOVA tests were used to compare the groups in terms of quantitative variables. A p-value of < 0.05 was considered statistically significant.

## Results

Out of 60 subjects, 56 completed the trial, and no patient experienced any adverse event. There were two dropouts in the curcumin group and two in the placebo group. All participants who dropped out did so due to personal reasons. Except for age, there was no significant difference in baseline characteristics including marital status, level of education, occupation, use of drugs affecting liver and blood lipids, sex, weight, BMI, WC, blood pressure (BP, FBG, total cholesterol, TG, HDL-C, LDL-C, AST, ALT, fibrosis, and hepatic steatosis between the two groups at baseline (Table 1).

**Table 1:** Comparison of baseline characteristics between curcumin and placebo groups.

	**Curcumin**	**Placebo**	***P*-value^#^**
N	28	28	
Gender (M/F)	17/11	11/17	0.109
Age (years)	43.92 ± 8.74	50.35 ± 9.44	0.011
Marital (married/single)	28/0	27/1	1.000
Education (Less than high school/ High school/ College education) (*n*)	8/12/8	13/11/4	0.277
Job (Freelance/ Employee/ Unemployed/ Retired)	13/6/9/0	10/4/11/3	0.262
Weight (kg)	82.72 ± 13.48	79.59 ± 8.81	0.309
BMI (kg/m^2^)	28.95 ± 3.24	29.28 ± 3.30	0.704
WC (cm)	104.98 ± 7.20	103.64 ± 7.44	0.497
BP (cmHg)	121.09 ± 15.51	121.11 ± 6.11	0.996
ALT (U/L)	40.03 ± 24.51	34.71 ± 18.43	0.363
AST (U/L)	34.50 ± 13.57	32.92 ± 13.27	0.663
FBG (mg/dl)	97.57 ± 18.72	99.35 ± 12.66	0.678
HDL-C (mg/dl)	46.03 ± 10.00	46.67 ± 10.01	0.811
LDL-C (mg/dl)	102.02 ± 32.23	105.59 ± 37.64	0.705
TC (mg/dl)	187.85 ± 37.46	193.57 ± 36.42	0.565
TG (mg/dl)	199.07 ± 87.13	189.96 ± 76.83	0.680
dB	299.71 ± 46.55	302.21 ± 42.07	0.834
kPa	5.42 ± 1.74	5.24 ± 1.29	0.672
Fatty liver stage	2.46 ± 0.88	2.35 ± 0.91	0.657
Steatosis	62.17 ± 25.17	62.75 ± 24.27	0.931
Fibrosis	1.67 ± 1.05	1.35 ± 0.67	0.181
Metformin	1.96 ± 0.18	1.92 ± 0.26	0.561
Statins	1.89 ± 0.31	1.85 ± 0.35	0.693

^#^Obtained from independent sample t-test or chi-squared test. BMI, body mass index; WC, waist circumference; BP, blood pressure; ALT, alanine aminotransferease; AST, aspartate aminotransferase; FBG, fasting blood glucose; HDL-C, high-density lipoprotein cholesterol; LDL-C, low density lipoprotein cholesterol; TC, total cholesterol; TG, triglyceride; dB, decibel; kPa, kilopascal.

No significant differences were found in dietary intake and physical activity of the groups except for protein intake, omega-6, and selenium (Table 2).

**Table 2:** Nutritional intakes and physical activity and their changes in the treatment and control groups at baseline, after 6 weeks, and after 12 weeks.

	**Curcumin group (*****n** =* **28)**				**Placebo group (*****n** =* **28)**		
**Variable**	**Baseline**	**Week 6**	**Week 12**	***P*** **-value** ^#^	**Baseline**	**Week 6**	**Week 12**	***P*** **-value** ^#^	***P*** **-value** ^*^
**Energy (kcal/d)**	1697.26 ± 664.08	1741.13 ± 646.33	1714.04 ± 642.18	0.775	1542.70 ± 629.37	1500.90 ± 570.04	1519.52 ± 525.57	0.625	0.220
**Carbohydrate (g/d)**	233.68 ± 96.54	241.48 ± 92.00	235.76 ± 94.39	0.739	211.75 ± 98.68	206.37 ± 110.58	212.20 ± 104.18	0.629	0.299
**Protein (g/d)**	79.17 ± 35.57	75.78 ± 31.08	77.44 ± 34.31	0.700	60.43 ± 18.05	60.61 ± 18.81	59.51 ± 18.48	0.912	0.013
**Fat (g/d)**	54.12 ± 20.00	56.69 ± 21.65	55.95 ± 19.95	0.410	54.33 ± 25.49	52.50 ± 15.76	53.15 ± 14.63	0.828	0.647
**Cholesterol (mg/d)**	220.62 ± 142.30	236.73 ± 163.44	243.15 ± 168.25	0.567	201.56 ± 113.22	211.34 ± 110.78	223.63 ± 129.61	0.441	0.530
**Fiber (g/d)**	28.16 ± 16.69	27.57 ± 10.15	28.86 ± 13.56	0.852	22.56 ± 11.81	24.44 ± 18.52	22.33 ± 13.83	0.486	0.145
**SFA (g/d)**	14.94 ± 6.28	15.29 ± 6.25	15.15 ± 6.42	0.875	16.49 ± 6.68	15.32 ± 4.67	15.08 ± 4.77	0.330	0.725
**MUFA (g/d)**	20.74 ± 8.19	21.26 ± 8.42	21.51 ± 7.94	0.552	19.79 ± 9.74	19.43 ± 6.32	19.60 ± 5.77	0.955	0.425
**PUFA- w6 (g/d)**	0.55 ± 0.32	0.11 ± 0.28	0.15 ± 0.31	0.613	0.80 ± 0.65	0.06 ± 0.16	0.04 ± 0.15	0.692	0.037
**PUFA- w3(g/d)**	0.20 ± 0.49	0.57 ± 0.32	0.55 ± 0.39	0.936	0.02 ± 0.08	0.69 ± 0.42	0.74 ± 0.54	0.510	0.076
**Vitamin E (mg/d)**	13.39 ± 4.45	14.37 ± 5.12	15.24 ± 5.07	0.011	11.92 ± 3.63	13.20 ± 6.76	12.84 ± 5.11	0.256	0.183
**Vitamin C (mg/d)**	82.46 ± 51.26	88.07 ± 40.82	89.43 ± 58.11	0.660	80.44 ± 73.45	97.63 ± 136.63	101.62 ± 149.38	0.258	0.783
**Zinc (mg/d)**	10.87 ± 5.78	10.84 ± 4.87	11.06 ± 5.12	0.906	9.01 ± 3.44	9.15 ± 3.16	9.06 ± 3.48	0.949	0.103
**Selenium (mg/d)**	100.15 ± 45.52	97.58 ± 42.36	99.50 ± 42.74	0.882	79.10 ± 29.37	79.00 ± 28.63	77.42 ± 26.83	0.899	0.027
**physical activity**	31.51 ± 5.61	32.03 ± 5.56	31.78 ± 5.07	0.548	30.61 ± 4.29	32.20 ± 3.71	31.76 ± 4.10	0.055	0.835

^**#**^Obtained from paired t-test. ^*^Obtained from analysis of covariance (ANCOVA), the mean values of outcomes were compared between groups, and adjustment was made for the baseline values of compared outcomes. SFA, saturated fatty acid; MUFA, monounsaturated fatty acid; PUFA, polyunsaturated fatty acid.

Consumption of phytosomal curcumin significantly reduced steatosis (*p* = 0.002), fibrosis (*p* = 0.027), and the stage of steatosis (*p* = 0.001) in the phytosomal curcumin group compared to the placebo group (Table 3). Even in some patients, the use of phytosomal curcumin, although it did not change the stage of steatosis, reduced the proportion of steatosis.

**Table 3:** Comparison of anthropometry, biochemical parameters, and FibroScan factors before and after the study.

	**Curcumin group (*****n =*** **28)**				**Placebo group (*****n =*** **28)**				**Between-group changes**
**Variable**	**Baseline**	**Week 12**	***P*** **-value** ^#^	**Mean difference** ±**SE**^a^	**Baseline**	**Week 12**	***P*** **-value** ^#^	**Mean difference** ±**SE**	***P*** **-value** ^*^
**Weight (kg)**	82.72 ±13.48	81.1 ± 12.59	0.005	−1.62 ± 0.53	79.59 ± 8.81	79.15 ± 8.74	0.339	−0.44± 0.45	0.167
**BMI (kg/m** ^**2**^ **)**	28.95 ± 3.24	28.36 ± 2.93	0.005	−0.58 ± 0.19	29.28 ± 3.30	29.14 ± 3.42	0.385	−0.14 ± 0.16	0.067
**Waist (cm)**	104.98 ± 7.20	101.67 ± 6.74	0.002	−3.30 ± 0.95	103.64 ± 7.44	104.19 ± 7.18	0.348	0.55 ± 0.57	0.001
**BP (mm/Hg)**	121.09 ± 15.51	117.44 ± 14.17	0.024	−3.64 ± 1.51	121.11 ± 6.11	123.85 ± 6.43	0.041	2.74 ± 1.27	0.001
**FBG (mg/dl)**	97.57 ± 18.72	99.64 ± 20.06	0.377	0.01 ± 0.02	99.35 ± 12.66	102.53 ± 14.78	0.148	0.02 ± 0.01	0.610
**Chol (mg/dl)**	187.85 ± 37.46	184.35 ± 33.23	0.475	−3.50 ± 4.83	193.57 ± 36.42	194.94 ± 40.91	0.772	1.37 ± 4.70	0.344
**TG (mg/dl)**	199.07 ± 87.13	186.03 ± 68.38	0.430	−13.03 ± 16.25	189.96 ± 76.83	162.92 ± 72.51	0.003	−27.03 ± 8.31	0.230
**LDL (mg/dl)**	102.02 ± 32.23	100.25 ± 36.61	0.691	−1.77 ± 4.41	105.59 ± 37.64	115.07 ± 41.48	0.049	9.47 ± 4.58	0.073
**HDL (mg/dl)**	46.03 ± 10.00	48.92 ± 12.61	0.289	2.89 ± 2.67	46.67 ± 10.01	48.25 ± 11.88	0.418	1.57 ± 1.91	0.743
**ALT (U/L)**	40.03 ± 24.51	34.35 ± 16.33	0.119	−5.67 ± 3.52	34.71 ± 18.43	30.37 ± 13.63	0.070	−4.33 ± 2.30	0.628
**AST (U/L)**	34.50 ± 13.57	31.89 ± 9.58	0.310	−2.60 ± 2.51	32.92 ± 13.27	31.25 ± 8.59	0.321	−1.67 ± 1.65	0.980
**dB**	299.71 ± 46.55	269.14 ± 52.66	0.001	−30.57 ± 7.90	302.21 ± 42.07	304.75 ± 41.37	0.794	2.53 ± 9.59	0.003
**kPa**	5.42 ± 1.74	4.81 ± 1.71	0.001	−0.60 ± 0.16	5.24 ± 1.29	5.27 ± 1.31	0.910	0.02 ± 0.21	0.028
**Steatosis**	62.17 ± 25.17	44.53 ± 30.43	0.001	−17.64 ± 3.68	62.75 ± 24.27	64.32 ± 24.43	0.771	1.57 ± 5.34	0.002
**Stage**	2.46 ± 0.88	1.64 ± 1.25	0.001	−0.82 ± 0.15	2.35 ± 0.91	2.46 ± 0.96	0.611	0.10 ± 0.20	0.001
**Fibrosis**	1.67 ± 1.05	1.39 ± 0.95	0.180	0.28 ± 0.11	1.35 ± 0.67	1.57 ± 1.06	0.184	−0.21 ± 0.15	0.027

^a^Standard error. ^#^Obtained from paired t-test. ^*^Obtained from analysis of covariance (ANCOVA), the mean values of outcomes were compared between groups, and adjustment was made for baseline values of compared outcomes.

Phytosomal curcumin was also able to significantly reduce WC (*p* = 0.001) and blood pressure (*p* = 0.001) compared to the placebo group (Table 3). At the end of the study, weight and BMI did not significantly decrease compared to the placebo group (*p* = 0.167, *p* = 0.067).

Moreover, 12 weeks of curcumin use could not significantly reduce liver enzymes and lipid profiles compared to the placebo group (*p* > 0.05). Conversely, curcumin consumption could not significantly reduce FBG compared to the placebo group (*p* = 0.610).

## Discussion

NAFLD is one of the most common chronic liver disorders in the world (1). In this study, our findings are of great clinical importance because only one clinical trial evaluated the efficacy of curcumin on NAFLD by FibroScan (40). This clinical trial showed that phytosomal curcumin had positive effects on fatty liver even at low doses. On the contrary, phytosomal curcumin consumption has important implications for managing NAFLD, which is a public health concern worldwide (30).

The present study showed that using phytosomal curcumin compared to placebo significantly improved fibrosis and steatosis symptoms in patients with NAFLD. In a study by Saadati et al. (40) the use of 1,500 mg of curcumin in patients with NAFLD could not significantly improve fibrosis and steatosis compared with the placebo group (40). Despite its many properties, curcumin has low bioavailability due to low intestinal absorption and rapid metabolism (41). The formulation of curcumin with phospholipids in the form of phytosomes increases the bioavailability of curcumin by improving intestinal absorption and metabolic stability (42), which might explain the different findings of this study compared with the study by Saadati et al. (40) in which a higher dose of curcumin in the crude form was used (40). To explain our findings regarding the beneficial effects of curcumin on fibrosis and steatosis, it should be mentioned that there is an association between oxidative stress, lipid peroxidation, and liver damage (43). Insulin resistance is a major pathophysiological factor in metabolic syndrome, which is independently associated with fatty liver (44). Therefore, curcumin can have beneficial effects on non-alcoholic fatty liver by controlling insulin resistance (45). Conversely, curcumin inhibits NF-κB activation; reduces downstream induction of ICAM-1, COX-2, and MCP-1; and decreases intrahepatic gene expression of monocyte chemoattractant protein-1, of CD11b, of procollagen type I, and on the tissue inhibitor of metalloprotease-1, leading to the mitigation of the development and progression of hepatic inflammation and fibrosis (46). In addition, curcumin enhances the activity of antioxidant enzymes, such as superoxide dismutase (SOD) and glutathione peroxidase (GSH), by scavenging free radicals (26). Thus, it appears that curcumin through the reduction of stress oxidative and inflammation plays a favorable role in liver diseases such as NAFLD.

In this study, the consumption of 250 mg/day of phytosomal curcumin by the participants in the phytosomal curcumin receiving group did not lead to a significant reduction in liver enzymes compared with the placebo group. However, according to the study by Panahi et al. (46) the consumption of 1,000 mg/day of phytosomal curcumin for 8 weeks significantly reduced liver enzymes (46). Conversely, according to the study by Mirhafez et al., the consumption of 250 mg/day of phytosomal curcumin for 2 months could not improve liver enzyme levels (47). The results of a systematic review study by Mansour–Ghanaei et al. (48) showed that curcumin in doses above 1,000 mg/day for a certain period of time can exert potentially beneficial effects on liver enzymes (48). Curcumin may improve hepatic steatosis and prevent the progression of fatty liver disease by inhibiting the synthesis of fatty acids and the biosynthesis of unsaturated fatty acids such as stearic acid, oleic acid, and linoleic acid (49). Although liver enzymes are elevated in most patients with NAFLD, relying on liver enzymes to diagnose and monitor disease progression may be misleading and unreliable because they can fluctuate throughout the course of the disease. The patient may also be in the final stages of fatty liver (cirrhosis), but the levels of these enzymes are normal (50). In this study, in comparison to the placebo group, both fibrosis and steatosis were significantly reduced in the phytosomal curcumin receiving group, although it had no significant effects on the liver enzymes. These contradictory results suggest that more studies are needed to show the effects of phytosomal curcumin on liver status. These results may indicate that phytosomal curcumin is more effective with higher doses and longer duration of use.

In the present study, the phytosomal curcumin receiving group resulted in a significant reduction in blood pressure compared to the placebo group. Hadi et al. (51) in their meta-analysis showed that taking curcumin for more than 12 weeks could have beneficial effects on blood pressure (51). Curcumin in high doses lowers blood pressure by inducing endothelial nitric oxide synthase (eNOS) protein expression and increasing antioxidant capacity through restoring glutathione (GSH) and a decrease in the overproduction of reactive oxygen species (ROS) (52, 53). Curcumin can improve the relaxation response of endothelial vessels to acetylcholine as well as increase the bioavailability of NO, and it also induces the expression of several antioxidant enzyme genes by the activation of the Nrf2-antioxidant response element signaling pathways (54–56).

In this study, supplementation with phytosomal curcumin resulted in a significant reduction in waist circumference compared with the placebo group, but it could not reduce weight and BMI. The results of a meta-analysis by Jafari Rad et al. (57) with 8 studies did not support the antiobesity properties of curcumin, while the results of a meta-analysis by Mousavi et al. (58) with 11 studies showed that curcumin consumption improves anthropometric indices. The reason for this discrepancy may be the condition of the patients. In Mousavi et al. (58) study, people with several different diseases were examined, while in Jafari Rad et al. (57) study, only patients with fatty livers were examined; therefore, the findings of Jafari Rad et al. (57) study may be influenced by the metabolic status and the context of the disease. On the contrary, in Mousavi et al. (58) meta-analysis, the articles were traversed with a long intervention period, while in Jafari Rad et al. (57) study, most trials had an intervention period of < 8 weeks, which is not sufficient to study the antiobesity effects of curcumin. Therefore, to better evaluate the effectiveness of curcumin on anthropometric indices, it is recommended to conduct more trials with longer intervention duration and different doses and types of curcumin. Nevertheless, it has been reported that curcumin can inhibit obesity by reducing the production of inflammatory compounds (59, 60). One possible mechanism of the impact of curcumin on anthropometric indices in cells and animals is accompanied by the suppression of peroxisome proliferator-activated receptor gamma and cytosine-cytosine-adenosine-adenosine-thymidine expressions (61, 62). Curcumin can also increase energy expenditure by increasing the production of adenosine triphosphate (ATP) and increasing AMP-activated protein kinase (AMPK) activity (63–65). Curcumin can affect obesity through hormones. Moreover, curcumin can lower leptin and increase adiponectin levels, thereby modulating appetite and energy homeostasis (66). Conversely, overproduction of the hormone adiponectin by activating AMPK can increase glucose utilization and oxidation of fatty acids, which facilitates weight loss (62, 67). In addition, because high levels of the stress hormone cortisol cause central obesity (68, 69), curcumin reduces the activating enzyme known as 11β-hydroxysteroid dehydrogenase type 1 (11β-HSD1) (68, 70). Curcumin can also cause weight loss by suppressing proinflammatory cytokines such as monocyte chemoattractant protein-1 (MCP-1), tumor necrosis factor-alpha (TNF-α), and plasminogen activator inhibitor type-1 (PAI-1) (65, 71).

Although it has been observed that curcumin has antidiabetic and lipid-modifying properties (26, 46), the results of this study showed that curcumin consumption did not lead to a significant reduction in profile lipids and FBG. The results of Mirhafez et al. (47) study with 250 mg of curcumin for 2 months on lipid profile and FBG are in line with our trial (47). The results of a meta-analysis of Jalali et al. (26) (*n* = 9) showed that the consumption of more than 500 mg/day of curcumin in patients with fatty liver has a positive effect on FBG and some lipid factors (LDL-C, TC) (26). Therefore, curcumin may have a better effect on FBG and lipid profile at higher doses, which explained the inconsistency in the results of our study with the recent meta-analysis (26).

In this study, the mean intake of selenium was significantly different between the study groups. We conducted further analyses to adjust its potential impact; however, the results remained unchanged (data were not shown). Indeed, although the selenium intake was significantly different between the groups, the differences were minor. In addition, as mentioned above, patients who received antioxidant supplementation such as vitamin E and selenium were excluded from the study. Therefore, although it has been shown that selenium supplementation can effectively alleviate metabolic disorders such as NAFLD (72), in our study, the difference in selenium intake did not account for the observed effects.

Although this study was one of the first double-blind, randomized clinical trials to investigate the effect of phytosomal curcumin as a natural and available herbal medicine on NAFLD using FibroScan, some limitations of the study should be noted. The sample size was small, and future studies are needed to be performed with a larger sample size. Conversely, we used a single dose of phytosomal curcumin, which makes it difficult to draw conclusions concerning the dose-dependent effects of phytosomal curcumin on fatty liver. In addition, not all participants had elevated transaminase levels at baseline, precluding the possibility of a precise assessment of the effects of supplementation on liver enzymes and function. Finally, although the patients were randomized and classified based on gender and steatosis status, the mean age was significantly different between the study groups at baseline. However, all patients were categorized as adults, and no significant physiological and clinical differences could be observed between the two groups, which can affect the NAFLD status among them.

## Conclusion

This double-blind, placebo-controlled clinical trial indicated that the daily intake of 250 mg phytosomal curcumin for 12 weeks could significantly improve liver fibrosis and steatosis and also reduce blood pressure and waist circumference. In addition, phytosomal curcumin could not significantly reduce fasting blood sugar, lipid profile, liver enzymes, and some anthropometric indices. Further studies are warranted to confirm these findings and to discover the underlying mechanisms.

## Data availability statement

The raw data supporting the conclusions of this article will be made available by the authors, without undue reservation.

## Ethics statement

The studies involving human participants were reviewed and approved by the IR.MUI.RESEARCH.REC.1398.461. The patients/participants provided their written informed consent to participate in this study.

## Author contributions

All authors designed the study, involved in the data collection, analysis, drafting of the manuscript, read, and approved the final manuscript.
